# Orbitofrontal-sensory cortical interactions in learning and adaptive decision-making

**DOI:** 10.1016/j.tics.2025.10.017

**Published:** 2025-12-04

**Authors:** Rohan Rao, Hugo Six, Aurelio Cortese, Abhishek Banerjee

**Affiliations:** 1 Biosciences Institute, Newcastle University, Newcastle, UK; 2 Blizard Institute, Queen Mary University of London, London, UK; 3 Adaptive Decisions Laboratory, Department of Pharmacology, University of Oxford, Oxford, UK; 4 Department of Decoded Neurofeedback, ATR Computational Neuroscience Laboratories, Kyoto, Japan; 5 Department of Biomedical Engineering, Sungkyunkwan University, Suwon, South Korea; 6 Center for Neuroscience Imaging Research, Institute for Basic Science, Suwon, South Korea

## Abstract

The orbitofrontal cortex (OFC) is a hub for value-guided decision-making, linked reciprocally with both cortical and subcortical regions. While projections from sensory areas to the OFC – and vice versa – are known to support goal-directed learning, these projections have often been studied in isolation, and their joint effect remains poorly understood. Here, we revisit these circuits through a unifying computational framework. We propose that sensory cortices send compressed task knowledge to the OFC to build abstract task models, while OFC feedback provides teaching signals that reshape sensory representations within the cortical hierarchy. This bidirectional exchange equips sensory areas with cognitive functions that extend well beyond passive feature detection, with significant implications for our understanding of learning, cognitive models, and artificial neural networks.

## The orbitofrontal and sensory cortical circuit in learning

When trying a new café during a morning commute, a rich, flavourful coffee will encourage you to return the next day, whereas a bland one will ensure that you will not. This everyday example illustrates the essence of reinforcement-guided learning (RL) (see Glossary), an effective framework for modelling learning processes [1,2]. Among the brain’s critical contributors to RL is the orbitofrontal cortex (OFC) [3–5], long recognised for its role in value computations and task-state representation [6–8]. Damage to the OFC impairs critical components of RL, including credit assignment and response-outcome mappings [9,10]. These computations depend on the OFC’s coordinated interaction with several cortical and subcortical brain areas, notably the hippocampus and striatum [11,12]. However, only recently have we begun to unearth the importance of sensory areas’ interactions with OFC.

Sensory cortices filter, represent, and relay sensory information, operating within a hierarchically organised system often known as the sensory hierarchy. These cortices maintain reciprocal connections with the OFC, supporting the bidirectional exchange of information [13]. Notably, the OFC is among the few frontal regions receiving direct anatomical input from all five sensory modalities [14], enabling multisensory information integration, thought to be necessary to form generalisable value representations [13]. Intriguingly, recent studies have challenged the traditional view of sensory cortices as mere feature detectors, revealing they may accommodate specific cognitive functions such as attention [15], working memory [16], tracking perceptual uncertainty [17], and active feature selection [18]. Human neuroimaging studies have even reported value-related coding in sensory areas that may depend on OFC input during RL tasks [19,20].

Despite these findings, research has progressed mainly along parallel tracks - either examining how sensory processing supports OFC function or how OFC modulates sensory functioning - largely overlooking their interaction. This paper aims to re-synthesise our current understanding of OFC–sensory cortical interactions during RL in light of new evidence for cognitive computations harboured within sensory cortices. We begin by outlining the anatomical connections between OFC and sensory cortices, then evaluate the roles of ‘sensory to OFC’ and ‘OFC to sensory’ pathways. Finally, we propose a conceptual framework in which reciprocal interactions jointly enable efficient RL.

## Anatomy of OFC’s connectivity with sensory cortices

The anatomical organisation of the OFC and its subregions’ contributions to reward-guided decision-making have been extensively reviewed [6,21,22]. OFC occupies areas 10,11,12,13,14 [23,24] and comprises highly interconnected subregions [25]. Barring area 13a, which is agranular, primate OFC is primarily granular, with granularity increasing along a posterior-to-anterior gradient [26]. Given the broad functional and cytoarchitectural similarities between non-human primates (NHPs) and human OFC, findings from NHP studies are often viewed as applicable to human OFC [27], and we draw on them as evidence for human OFC functions. However, cross-species comparisons are not straightforward: while value signals during RL are found in human ventromedial prefrontal cortex/medial OFC (vmPFC/mOFC), they are located in central OFC in monkeys, despite cytoarchitectural and connectivity differences between these regions [28]. Thus, the precise homology between human and primate OFC remains a topic of active debate [28]. As the rodent OFC is agranular, some have argued that it is homologous to the agranular region of the human OFC (area 13a) [27]. In both species, this region shares reciprocal connections with sensory cortices across all modalities and encodes reward value [29], with lesions impairing extinction learning [30]. However, rodents lack a clear homologue of the human granular OFC, and the translational relevance of rodent PFC studies to humans is debated (see [31,32] for detailed reviews). This debate is more contentious than for NHP–human comparisons, likely due to greater phylogenetic distance. Nevertheless, animal models are essential for advancing our understanding of PFC function, as stressed by many [31,32]. Throughout this paper, we indicate when findings from rodent OFC studies are used to support our claims.

**Figure 1:**
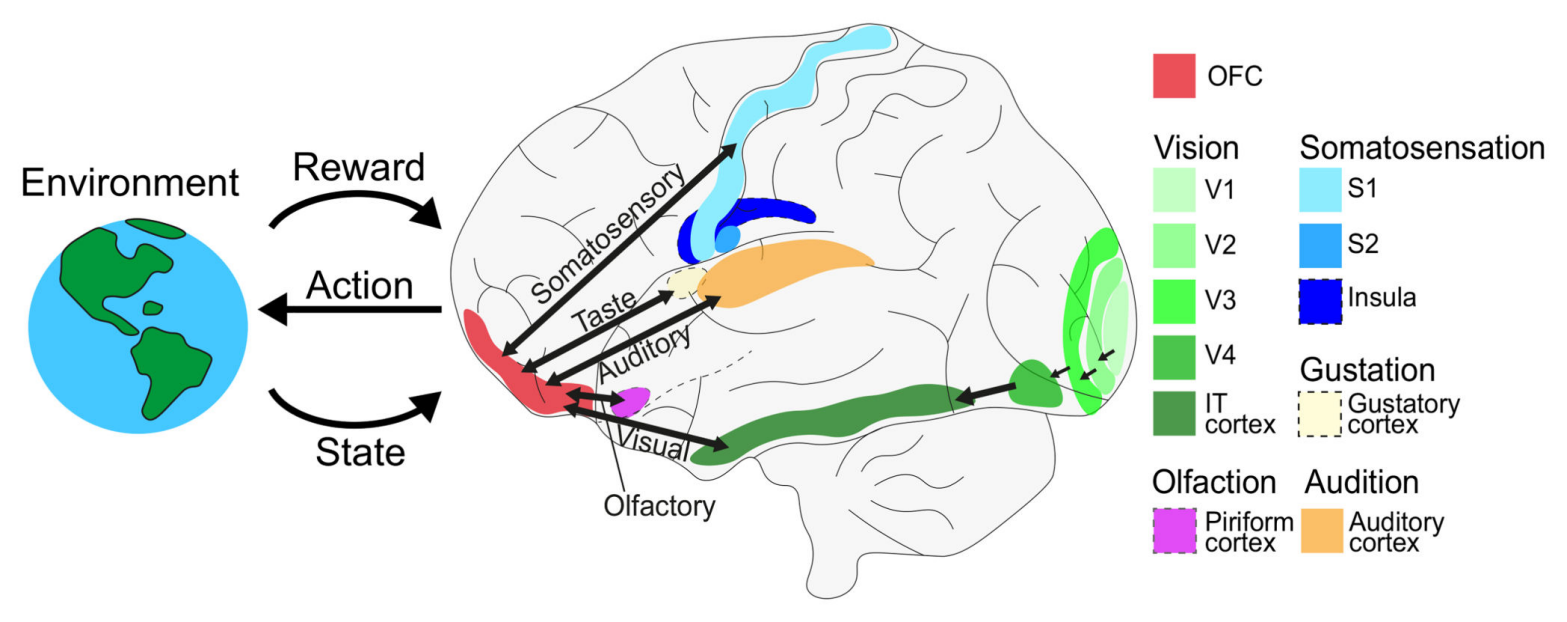
Major anatomical connections between sensory cortices and the orbitofrontal cortex (OFC). A lateral view of the major anatomical connections between orbitofrontal and sensory cortices in the human brain. The OFC shares reciprocal anatomical connections with sensory and association cortices from all sensory modalities [14]. These cortices include (i) primary and secondary somatosensory cortex (S1 and S2), somatosensory insula and the frontal operculum [14]; (ii) primary gustatory cortex for gustation [14]; (iii) piriform cortex, entorhinal cortex and anterior insula for olfaction [14,35]; (iv) the superior temporal gyrus, including the auditory rostral belt and parabelt for audition [35]; (v) the inferior temporal cortex (IT), perirhinal cortex and the superior temporal sulcus for vision [34]. As granularity increases from the OFC’s posterior-to-anterior gradient, multimodal sensory inputs also become sparser [26]. These anatomical connections enable the OFC (in particular, posterior OFC) to integrate multimodal sensory information from the environment about current states and rewards to support action selection during reinforcement learning tasks. Anatomical connectivity in this context is based on tracer studies in non-human primates. Dotted outlines indicate interior brain areas. Abbreviations: V1, primary visual cortex; V2−V4, higher visual cortices.

In humans and NHPs, the OFC maintains reciprocal connections with a mixture of primary and higher sensory cortices across modalities [13,33] (Figure 1A). These inputs are more prominent in the lateral OFC (IOFC) than the mOFC [25]. The OFC is considered a higher-order olfactory and gustatory cortex, receiving strong inputs from the respective primary sensory areas. It also connects with primary and higher somatosensory cortices, while visual and auditory inputs arise predominantly from association areas, such as the ventral visual stream and belt/parabelt auditory regions [34,35]. These projections to the OFC spanning heterogeneous levels of sensory hierarchy have been hypothesised to convey stimulus identity signals [33], such as object and face recognition from the inferior temporal, fusiform, and rhinal cortices [34]. We discuss the evidence for this hypothesis and other functions of feedforward sensory to OFC projections in the next sections.

Interestingly, posterior OFC receives more inputs from primary sensory areas, while anterior OFC receives more from association areas [14]. Though some studies report structural and functional OFC connectivity with primary visual and auditory cortices in humans [20,33,36–38], these are not consistently supported by tracer studies and are therefore not the focus here. Notably, based on corticocortical interconnectivity findings, the OFC overlaps with broader ‘orbital’ and ‘medial’ prefrontal networks [25] implicated in sensory integration and emotional regulation hubs, respectively [39].

## Sensory projections to OFC

What is the functional role of bottom-up sensory inputs to OFC? This question has received considerable attention across species in recent decades [40–42]. Here, we first review the prevailing view in systems neuroscience that these inputs merely provide the OFC with sensory information during RL. We then update this perspective in light of recently discovered cognitive functions of sensory cortices and emerging theories on the OFC’s role in representing latent task states.

### How do sensory inputs classically support OFC value computations?

**Figure 2:**
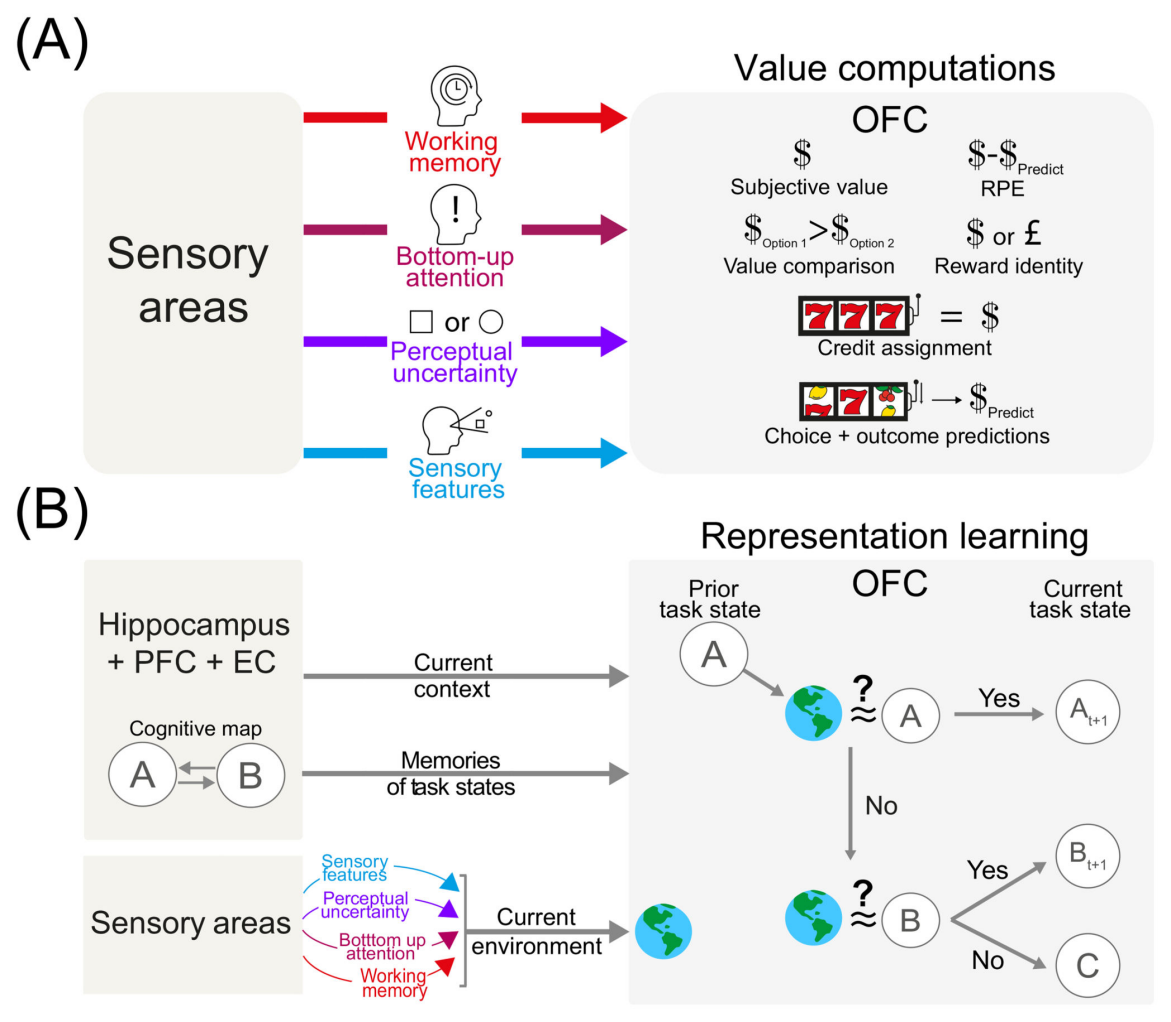
Contribution of sensory inputs to value computation and representation learning in the orbitofrontal cortex (OFC). (A) Schematic illustrating how the OFC may use higher-order cognitive information from sensory cortices to perform value computations. The OFC may use perceptual uncertainty signals [70] to modulate the strength of reward identity, credit assignment and outcome prediction codes [72,74]. Bottom-up attention and salience signals [15] highlight relevant stimuli and choices during reward learning [57], such as a slot machine catching your eye in a casino. Sensory working memory [67] can be used to compare sensory features of perceptually similar stimuli that predict reward, facilitating value comparison and credit assignment. (B) Proposed mechanism for representation learning in the OFC. Sensory inputs filtered through cognitive computations are integrated with contextual and memory information to compare perceived environments to a prior state ‘A’ [76]. If dissimilar, the OFC checks for similarity with previously experienced states (e.g., state ‘B’), which are stored in the hippocampus, entorhinal cortex (EC), and other prefrontal cortex (PFC) regions [11,76,82,83]. If no match is found, the OFC defines a novel state ‘C’ [76] with an associated exploration bonus [3] for new options, supporting adaptive learning. Abbreviation: RPE, reward prediction error.

RL unfolds as a series of interactions between an agent and its environment, typically through trial-and-error (Figure 1A). First, the agent estimates the current state from environmental inputs and its latent knowledge (value expectation, or an internal model of the world). It then selects an action that maximises future returns. The agent finally updates its latent knowledge based on the outcome (rewarding, aversive or neutral). From a computational perspective, the human OFC appears to be a critical component in many RL algorithms (Box 1), involved in distributed value computations and state representations (Figure 2A). These value computations include encoding subjective value [7], reward prediction errors (RPEs, in lOFC) [19], value comparisons (mOFC) [9,43], reward identity (lOFC) [41], and credit assignment (lOFC) [9,20]. Although these computations serve distinct roles within RL, many of them depend on inputs from sensory cortices to the OFC.

Sensory cortices are thought to provide the OFC with essential information about environmental features, task uncertainty, and volatility inferred from stimuli statistics – enabling context-sensitive value encoding [42]. For instance, when we enjoy a cup of coffee, OFC neurons are thought to compute its subjective value by integrating sensory inputs (e.g., flavour, colour, and texture) with cognitive signals from subcortical regions. These include emotional inputs from the amygdala (e.g., stress [44]), physiological states from the hypothalamus (e.g., hunger [45]), and reward or RPE information from the nucleus accumbens, ventral striatum and dopaminergic ventral tegmental area (VTA) pathways [12,46,47]. Additionally, contextual and memory-related information from the subcortical hippocampus and prefrontal cortical areas (e.g., brand reputation [11,48,49]) reaches the OFC. Intriguingly, rodent studies have shown that OFC population-level value responses may emerge from subpopulations of ‘pessimistic’ and ‘optimistic’ neurons that under-estimate and overestimate, respectively, the utility and probability of reward [50], a mechanism that could extend to primates.

The importance of sensory inputs to OFC in value-based decisions has been illustrated through chemogenetics silencing of perirhinal cortex projections to the OFC, which reduced sensitivity to reward magnitude and disrupted expected value computations [42]. As the perirhinal cortex is often considered an extension of the ‘ventral visual stream’ and is associated with object recognition, these findings suggest that the OFC integrates stimuli, or event, identity information with reward associations to inform choices.

The OFC transmits integrated value signals via local circuits that compute comparisons between options, and disrupting these signals (electrically or pharmacologically) impairs behaviour [4,43]. Its access to multimodal sensory inputs presumably allows integration of rewards across modalities – for example, combining gustatory and olfactory features of coffee into a unified scalar value [51]. Supporting this view, subpopulations of OFC neurons encode sensory-specific value [52], which may be combined to enable cross-modal value comparisons. Although value signals also exist in the dopaminergic system [47], the OFC stands out for its consistent value representations across tasks [53].

To support optimal decisions in RL, the lOFC assigns value to specific stimuli and actions – a process known as credit assignment [9,42]. This likely relies on dynamic interactions with sensory cortices, which can provide real-time contextual information [42]. When rewards are immediate, lOFC neurons could integrate sensory inputs with reward signals from the ventral striatum and dopaminergic systems [12,47]. For delayed rewards, it may draw on contextual and mnemonic information from the hippocampus, prefrontal cortex, and working memory [11,48,49,54]. Sensory inputs may thus modulate credit assignment strength in both cases, enabling flexible value comparisons across time and contexts.

### Sensory areas precompute task information to support OFC value computations

In value computations, projecting sensory information about observable environmental features to the OFC is a core function of sensory cortices. But they may also transmit higher-order cognitive signals, such as stimulus salience – how much a stimulus stands out – encoded via saliency maps [55] and population activity patterns [56]. This bottom-up attentional capture can influence prefrontal areas, including the OFC [57,58] (Figure 2A). For instance, OFC neurons have been shown to transiently encode the value of a luminance-changing cue even when it lacked predictive value [57], suggesting salience alone can modulate OFC activity. Since salient stimuli often signal potential rewards or punishments [59], they may accelerate OFC value coding [57,58] (Figure 2A). Consistent with this interpretation, rats’ ventral/lateral OFC neurons preferentially encode salient stimuli, regardless of reward association [60]. The OFC may improve credit assignment and facilitate learning in complex environments by amplifying salient inputs. However, it remains unclear whether salience inputs to the OFC come primarily from sensory cortices or other brain areas such as the mediodorsal thalamus and the inferior frontal gyrus [61,62].

Beyond bottom-up attentional capture mechanisms, sensory cortices also support sensory working memory [63–65]. Sensory neurons can maintain stimulus representations after offset through sustained activity, population coding, and oscillatory synchrony with the prefrontal cortex [16,64–66]. The fidelity of these representations can even predict behavioural performance during delayed discrimination tasks [67]. This function could be especially useful during credit assignment, where the OFC must discriminate between multiple stimuli or their combinations. While the hippocampus and PFC also provide working memory signals to the OFC [54], sensory cortex inputs may deliver more precise, real-time information about recent stimuli (Figure 2A).

When faced with noisy or ambiguous input, the brain must evaluate competing interpretations of the environment. A useful concept here is perceptual uncertainty, the inverse of how confidently an agent can identify the true signal. Perceptual uncertainty aligns well with the Bayesian perspective, in which probabilistic neural codes in sensory areas simultaneously encode the estimated identity (mean) and uncertainty (variance) of a stimulus [68]. Information about stimulus uncertainty and probability [69,70] could provide valuable information for downstream computations in regions like the OFC. Indeed, higher visual cortices, such as the inferior temporal cortex, have been shown to have enhanced connectivity with frontal cortices in trials with high perceptual uncertainty [70]. It is worth noting that the OFC itself is thought to track perceptual uncertainty as a scalar signal [71]. The OFC might further integrate perceptual uncertainty with other sources of uncertainty (e.g., reward probability [72]) to generate abstract belief or confidence signals, guiding action selection [73]. Confidence signals may also intrinsically modulate value computations in the OFC (Figure 2A). Indeed, one of our laboratories found that higher perceptual uncertainty corresponded to reduced value-related activity in the mOFC/vmPFC [74]. Relayed to the OFC, these sensory signals could refine outcome predictions for perceptually similar stimuli.

### How do sensory areas support task representations in the OFC?

While traditionally associated with value computations, a parallel research stream suggests that lOFC [11] and mOFC/vmPFC [75] also represent abstract task states within a non-spatial cognitive map (see [6,76]). Proposed by Tolman [77] and later supported by hippocampal studies [78], the cognitive map concept has been expanded to encompass abstract task structures [79,80]. A key study [7] found that mOFC selectively encoded task-relevant observable variables (in the current trial) and latent variables (relevant information in the previous trial) but not task-irrelevant variables, indicating its role in representing the current task state [8]. This abstract representation allows the OFC to guide behaviour via projections to the striatum, ACC, and lateral PFC [79,81], supporting RL, generalisation, and continual learning.

We propose that sensory cortices are central to the OFC’s ability to efficiently construct task-state representations – a process known as representation learning [76]. The OFC may compare incoming sensory inputs with stored task states in the hippocampus and entorhinal cortex [82,83]. If the input matches a known state, the OFC reuses and updates that memory; if not, it generates a new state. Algorithmically, this could involve sequential comparisons (Figure 2B). For instance, an agent currently in state ‘A’ might: (i) update their current state to ‘A_t+1_’ if the input is similar; (ii) switch to a known state ‘B’; or (iii) create a new state ‘C’. Sensory cortices may additionally compress and relay features like salience, working memory content, and perceptual uncertainty to support this process. Updated states can then be sent back to memory systems and other prefrontal areas, continuously recalibrating the brain’s internal cognitive map [11,82,83].

Our proposal aligns with models of hippocampal function that balance pattern separation and generalisation [84], and echoes recent computational models of working memory that invoke similar mechanisms [85]. We suggest that sensory cortices provide the OFC with precomputed, compressed task knowledge (stimuli identity, uncertainty, past stimuli, salience) that support the construction of task states [20,80,86]. While sensory cortices do not have access to the whole set of associations as the OFC or hippocampus might have, they can still reduce the dimensionality of this sensory input in a useful manner, that is, they can ‘compress’ task knowledge. This early-stage dimensionality reduction at the sensory level may facilitate representation learning in the OFC. This idea also aligns with recent theories of compositionality, which propose that both biological and artificial systems build complex representations by combining simpler, reusable components [87].

Recent research suggests that the OFC additionally encodes broader cognitive maps – representations of the relationships between task elements or states [88,89]. These maps are well suited for supporting model-based RL (Box 1) as they help predict how actions lead to state transitions. Cognitive maps (or meta-maps) likely span the OFC, the hippocampus, the entorhinal cortex and other prefrontal regions [48,82,83], though how this information is distributed across these regions remains under investigation. Notably, hippocampal-to-OFC theta oscillations may transmit state information, with the OFC preferentially encoding state-dependent values rather than states themselves [90]. Developing theoretical frameworks that reconcile OFC’s function in task-state representation and state-dependent valuation will be essential moving forward.

## OFC control signals to sensory areas support RL

Similar to bottom-up sensory to OFC projections, the function of top-down projections has been studied across species in the context of RL [19,91]. Intriguingly, these top-down projections may drive reward expectation [92], value-driven attentional capture [93], and coding remapping [19] directly within sensory areas. In the following sections, we review the evidence supporting these functions and discuss their implications for sensory processing during RL.

### OFC control signals improve perceptual processing in sensory areas

**Figure 3:**
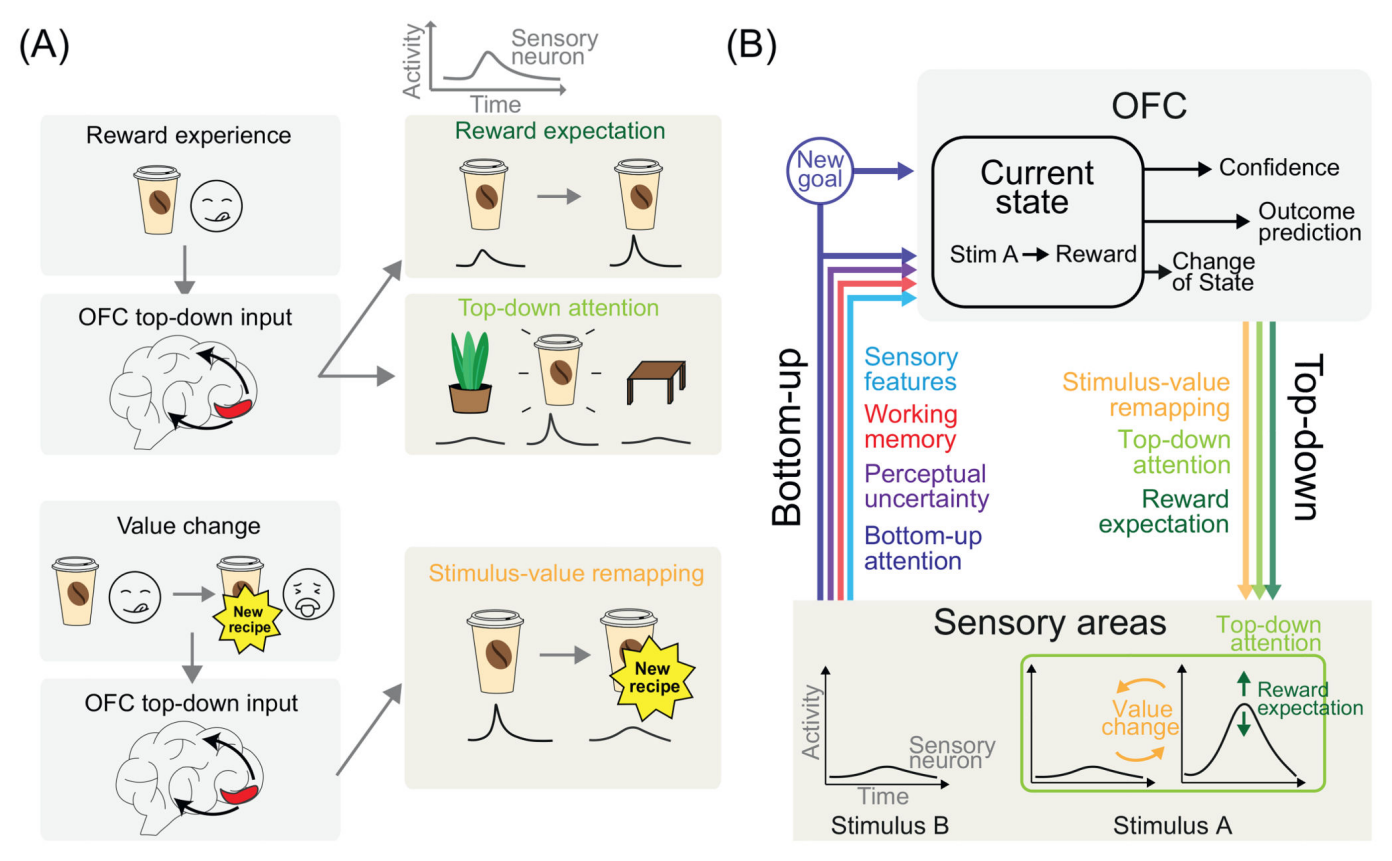
The orbitofrontal cortex (OFC)–sensory cortex interactions supporting reinforcement-based adaptive learning. (A) Following a rewarding experience (e.g., an enjoyable coffee), the OFC can engage two complementary top-down mechanisms. First, it sends a reward-expectation signal that enhances the encoding strength of sensory neurons for stimuli associated with reward [99]. Second, the OFC issues a goal-directed attentional signal [93] that increases sensory neuron responses to goal-relevant stimuli while filtering out irrelevant stimuli. Both mechanisms sharpen neuronal representations of relevant stimuli, resulting in enhanced perception and improved sensory acuity for task-relevant features. Otherwise, when a stimulus’ value changes abruptly (e.g., the coffee becomes unpleasant), the OFC can send top-down remapping signals to sensory cortices [19,91]. These signals dampen neural responses to the previously rewarding stimulus, supporting rapid behavioural adaptations. (B) In our proposed model, sensory cortices send multiple information streams to the OFC: sensory features help to construct OFC task states. Bottom-up attention directs the OFC to prioritise salient stimuli, which may trigger updates to task state or goals. Sensory working memory enables discrimination between similar stimuli (e.g., distinguishing task-relevant stimulus A from a recent but irrelevant stimulus B), improving outcome predictions. These predictions in turn drive top-down reward expectation and value-remapping signals backto sensory cortices. Perceptual uncertainty reflects the reliability of stimulus representations and informs OFC confidence estimates that modulate its outputs, enhancing signals under high certainty and decreasing signal strength when confidence is low. OFC remapping signals enable rapid neural re-tuning during changes in task context.

Value signals can enhance perception [20,94], with high-reward stimuli often more accurately discriminated than low-reward ones [95]. One mechanism may involve OFC-derived teaching signals that adjust gain or receptive fields in sensory cortices based on reward history or expectations [92] (Figure 3A). Rodent studies reveal modality-specific pathways for this effect. In vision, OFC projections to primary visual cortex (V1) somatostatin interneurons suppress responses to unrewarded stimuli [96]; in audition and olfaction, projections to excitatory and inhibitory neurons in primary auditory cortex (A1) and piriform cortex amplify responses to reward-associated stimuli [97,98]. These findings suggest conserved mechanisms by which the OFC can modulate higher visual (e.g., fusiform cortex [99]) and auditory regions (belt areas and superior temporal gyrus [100]) to support perception via value-based feedback. Note that OFC-to-primary visual and auditory cortex (A1, V1) projections appear unique to rodents. Complementary to reward expectation, value-driven attentional capture enhances perception across modalities: stimuli linked to reward or punishment reliably draw attention in both visual and auditory domains [101,102]. This is typically attributed to frontoparietal control networks, which modulate sensory cortices via long-range projections [103]. However, the OFC may also directly influence sensory cortices, strengthening task-relevant sensory representations via its own projections [93]. This mechanism could act synergistically with frontoparietal and midbrain (e.g., VTA) inputs [104] to sharpen task-relevant perception during RL (Figure 3A).

It is important to mention that the OFC connects with higher-order visual and auditory cortices, but with both primary and higher cortices in other sensory modalities. This anatomical pattern suggests that top-down OFC signals may selectively enhance perception at the level of what is typically most relevant for behaviour, such as stimulus identity in vision (e.g., objects [99]) and audition (e.g., speech [100]). While in other modalities, these signals may additionally modulate feature-level discrimination. Since rodents exhibit direct projections from OFC to the primary visual [96] and auditory cortices [97], there may be evolutionary divergence in the role and granularity of OFC top-down control across species.

### OFC signals can remap value encoding in sensory areas

OFC projections to sensory cortices can enact value remapping, that is, signal abrupt changes in stimulus-outcome contingencies (Figure 3A) [19,91]. In one of our laboratories, we investigated these top-down ‘teaching’ signals using fMRI during a probabilistic tactile reversal learning task [21]. Following contingency reversal, we observed a transient increase in functional connectivity between the lOFC and ipsilateral reward-selective regions of primary somatosensory cortex (S1), which declined as participants adapted. Crucially, lOFC outcome-related activity preceded S1 responses during the reversal phase, consistent with lOFC-driven reconfiguration of sensory-reward representations to support flexible behaviour. Nevertheless, confirming the directionality of these signals will require causal methods such as Granger causality, transcranial stimulation, or neurofeedback-based approaches [105,106]. LOFC activity also decreases with learning expertise, decreasing as participants move from naïve to expert phases [19]. This mirrors findings that PFC engagement decreases as performance becomes more efficient [107], possibly reflecting a reduction in cognitive demands. One interpretation is that, during expert phases, reward-related responses become stable and selective in sensory areas such as S1 [19], the auditory belt [108], primary gustatory cortex [109], and the inferior temporal cortex [110] – allowing these areas to assume greater value processing roles. This shift may offload computational burden from lOFC once stimulus-action mappings are well established [19]. Lesion studies nonetheless offer a nuanced picture: while OFC damage impairs reversal learning and increases lose-stay behaviour in probabilistic tasks [111], it has minimal effects in deterministic settings [112]. This suggests that OFC switch signals may be particularly relevant in uncertain environments in primates.

Complementing correlational findings in humans and primates, rodent studies offer causal evidence for OFC-driven value remapping in sensory cortices. In one study from one of our laboratories, mice performed a tactile Go/No-Go reversal learning task [91]. Following reversal, a fraction of value- and outcome-selective S1 neurons initially lost their selectivity but reacquired it with learning – unless IOFC→SI projections were silenced, which abolished both neural remapping and behavioural adaptation. Further analyses revealed that this top-down signal also remapped S1 reward-expectation selectivity and resembled a context-prediction error [113]. Such a signal is consistent with model-based and meta-RL frameworks (Box 1), as it supports generalisation by updating stimulus-outcome expectations even for unobserved stimuli following reversal [114,115].

## Closing the loop through a holistic RL framework of OFC and sensory interactions

Most RL research treats bottom-up sensory inputs to OFC and top-down OFC projections to sensory areas as separate processes. Mirroring this approach, we introduced their roles separately. But this division overlooks their dynamic interaction. In reality, bottom-up inputs can shape precise top-down signals, and vice versa. We now propose a closed-loop framework that captures how synergy between these pathways can benefit RL.

In our framework, sensory inputs help build and update accurate task-state representations in the OFC (Figure 3B), encoding all relevant variables, including predictions of prospective outcomes. These internal OFC states then guide precise reward expectations and attentional signals, which, in turn, enhance perception of task-relevant features. Such a reciprocal loop reduces perceptual uncertainty, sharpens neural representations, and supports adaptive behaviour by continuously aligning sensory processing with task demands [56].

Reversal learning is a second key context to discuss the importance of reciprocal OFC-sensory interactions in RL. After reversal, top-down OFC signals may remap outcome and expectation responses in sensory cortices. These areas, in turn, may integrate sensory-reward signals with higher-order cognitive information to deliver compressed, task-relevant inputs back to OFC for task-state reconstruction and comparison. Additionally, sensory inputs conveying perceptual uncertainty may modulate the strength of OFC feedback. In ambiguous contexts, lower decision confidence [73] could weaken or diffuse OFC signals, resulting in less precise modulation of sensory encoding that reflects uncertainty in credit assignment.

While several perspectives on OFC function in RL exist (Box 1), our framework emphasises the OFC’s role in constructing task-state representations within a broader ‘world model’ or cognitive map, concepts often linked to model-based and meta-RL. Our framework, however, does not exclude other algorithmic implementations such as model-free RL, which we suspect may be engaged differentially depending on task demands, behavioural strategies, or environmental context, potentially shaping top-down teaching signals. Although our focus is on OFC-sensory interactions in task-state representation, choice, and learning, we recognise the critical roles of other regions – including the hippocampus, entorhinal cortex, midbrain, and mesolimbic system - in supporting RL [53]. We thus position our proposal as one flexible component within a broader, distributed neural architecture that supports RL.

## Concluding remarks

The functional role of sensory cortices in cognition has undergone a profound shift. Once considered passive encoders of physical features – such as tonotopic [116], somatotopic maps [117], or visual receptive fields [118] – they are now recognised as active contributors to higher-order functions. This reappraisal carries important, yet underexplored, implications for how sensory projections influence executive regions, such as the OFC, during learning and decision-making.

We have highlighted how sensory cortices contribute to bottom-up attention [15], sensory working memory [16] and perceptual uncertainty [17], and how these signals, when projected to the OFC, can support value computations and representation learning. We also discussed how top-down OFC signals may enhance the encoding of task-relevant and reward-associated stimuli in sensory cortices, potentially improving perception and redistributing value computations from the OFC to sensory areas. While some of these ideas are speculative, the role of value coding in sensory cortices during RL is a promising direction for future research (see Outstanding questions). These insights extend beyond neuroscience (Box 1), suggesting that AI systems (which often neglect cognitive roles for sensory units) may benefit from incorporating such dynamics. While some mechanisms – like concept-specific units [119] – can arise in deep learning models, others, such as top-down modulation of sensory representations, are not easily captured by standard backpropagation. Whether OFC-like tuning shifts can be modelled in artificial systems remains an exciting challenge.

In sum, we proposed a framework in which reciprocal OFC-sensory cortex interactions dynamically support RL. Future work should test these bidirectional circuits through carefully designed experiments (see Outstanding questions). Ultimately, we advocate moving beyond simple ‘A-to-B/B-to-A’ models towards an integrative, systems-level understanding (‘A and B’) of the circuitry underlying value-guided behaviour.

## Highlights

Reciprocal connections between sensory cortices and the orbitofrontal cortex (OFC) support value computations and the construction of task states during reinforcement-guided learning.

We assess primary sensory cortical projections of task-relevant information to the OFC, including perceptual uncertainty, salience and sensory working memory, departing from classical theories of sensory processing.

OFC feedback projections to sensory areas instruct and enhance sensory representations of task-relevant stimuli.

We propose a synergistic model of closed-loop OFC–sensory cortical interactions that supports efficient learning in the human brain.

## Glossary

### Abbreviations

Bottom-up attentional capture: allocation of attention to a stimulus that is sufficiently salient, driven by the sensory properties of the stimulus that might differ from top-down attended features.
Cognitive map: the systematic organisation of physical or abstract entities and the relationship between them in artificial or biological neural systems.
OFC–sensory cortical interactions: the anatomical and functional direct and indirect connections between the orbitofrontal cortex and sensory cortices from all five sensory modalities.
Perceptual uncertainty: the degree of ambiguity in the agent's perception of stimuli in its environment, which can arise from various sources, including sensory noise, ambiguity in the environment, and uncertainty in the agent's internal models.
Reinforcement-guided learning (RL): a trial-and-error learning process shaped by rewarding and aversive outcomes.
Representation learning: learning a representation of the environment based on sampled stimuli that makes it easier to extract useful information according to the agent’s goal.
Sensory hierarchy: classical hierarchical organisation of sensory processing in the brain, where primary sensory areas receive thalamic projections of sensory information and encode sensory feature information before relaying this information to higher-order sensory areas involved in higher functions such as object recognition.
Sensory working memory: the storage and recall of sensory information by an artificial or biological neural system for a short duration after the relevant information is no longer observable.
Task-state representation: collection of observable and non-observable information necessary to predict decision outcomes. The transitions between task states constitute a Markov decision process that allows RL algorithms to solve the reward maximisation problem.
Value computations: the process by which the brain represents, updates and manipulates subjective values associated with stimuli or actions using sensory information, prior knowledge and goals.
Value remapping: update of the value or reward associated with a stimulus or action based on new information or experience, enabling the adaptation of behaviour to a changing environment.

## Box 1 RL and the OFC

RL provides a powerful framework to study decision-making. Yet, its neural implementation remains debated [53]. Here, we introduce several RL formalisms that could be supported by OFC–sensory interactions (Figure I).

Model-free RL involves learning a decision policy through trial-and-error without explicitly modelling environmental dynamics, aiming to maximise cumulative reward [2]. While the OFC may play a limited role in model-free learning [120], it contributes useful representations of reward values [7] and exploratory behaviour [3].

Model-based RL builds a model of the environment (i.e., state transitions) to guide decision-making [2]. The OFC supports this by encoding both current task states and higher-level abstracted task structure [88], binding sensory and latent information to form cognitive maps of task space [89].

Deep-RL leverages deep neural networks to handle high-dimensional state spaces [121]. Though originally applied to vision [122], recent work extends deep RL to model higher cognitive functions [123], including value encoding in the OFC via recurrent neural networks [124]. This architecture offers a compelling framework for modelling OFC–sensory interactions. For instance, bottom-up feature extraction through feedforward computation and top-down learning signals through backpropagation or other novel credit-assignment mechanisms such as prospective configuration [125].

Meta-RL allows agents to learn across tasks and timescales by updating meta-parameters through parallel reinforcement procedures [114,126]. These models learn ‘how to learn’ and generalise across contexts. Recurrent networks trained via meta-RL replicate key aspects of OFC dynamics during reversal learning in mice [115]. However, improved value discriminability shown by the model was not observed experimentally - possibly because learning computations shifted to sensory areas via OFC feedback.

Distributional RL extends standard RL by learning full reward distributions for state-action pairs rather than single expected values [127]. This captures variability in outcomes and supports more flexible decision-making. While direct evidence in OFC is limited, recent findings show OFC neurons may be tuned to stimuli near the tails of reward distributions [128].

Separately from RL, OFC may play roles in other cognitive frameworks, such as signalling ‘belief states’ within Bayesian inference frameworks [129]. Many of these models are not mutually exclusive, and studies have suggested they may even be used in parallel in certain tasks [53]. Thus, a possible reconciliation of these frameworks in the brain is that they are task and context-specific, with OFC, vlPFC, and frontopolar cortices potentially coordinating model arbitration [53].

## Outstanding questions

Are the mechanisms underpinning OFC teaching signals to sensory areas distinct across task space and task type? Are they conserved across species, and what might that signify?

What function do value representations in sensory areas play in value-guided decision-making?

Is value an abstract state in RL? If so, how can this be integrated into RL models?

How are value and task-state representations in OFC used to create teaching signals that cause plasticity changes in sensory areas?

Do unilateral and bilateral coupling measures between OFC and sensory cortices in human learning studies generalise across sensory modalities?

Is the underlying mechanism of top-down OFC teaching signals to sensory cortices consistent across sensory modalities?

What is the role of intrinsic value signals, for example, derived from affective computations?

What happens when value and sensory-OFC loops converge on the wrong dimension(s)?

Does silencing of salience, perceptual uncertainty, or working memory encoding neurons in sensory cortices alter OFC task representations?
